# The effect of nurse health literacy interventions on patient health literacy scores in specialty consultations: a quasi-experimental study

**DOI:** 10.1186/s12912-024-02447-1

**Published:** 2024-10-25

**Authors:** Angela McCaskill, Angel Gasch-Gallen, Jesica Montero-Marco

**Affiliations:** 1 Department of Physiatry and Nursing, Faculty of Health Sciences, University of Zaragoza, C/Domingo Miral, s/n 50009, Zaragoza, Spain; 2 Research Unit, Hospital Clínico Universitario Lozano Blesa, Avda. San Juan Bosco, 15, Zaragoza, 50009 Spain

**Keywords:** Health literacy, Health literacy strategies, Patient education, Standardization in patient education, Population health, Health literacy checklist

## Abstract

**Background:**

Patient health literacy (HL) affects health and wellbeing on both individual and population levels. The ability to receive, understand, manage and act upon health information can be positively influenced by nurses’ use of HL strategies. This study examined the relationship between nurses’ use of a HL checklist (intervention) and before and after patient HL scores, and the effects of frequency and types of strategies used in specialty consultations in Spain.

**Methods:**

This quasi-experimental, non-randomized study used the HLS_19_-Q12 to calculate HL scores for 149 patients. Calculations were performed both before and after a nursing intervention that consisted of using a HL checklist. Paired samples t-test assessed the difference between patient HL scores pre- and post-nurse intervention. Frequency analysis and Pearson correlation where used to examine frequencies of nursing HL strategies used and associations with HL scores.

**Results:**

The mean difference between the HLS_19_-Q12 scores before and after intervention was − 9.94, with a standard deviation of 11.50. There was a statistically significant effect of the intervention on HL score (t = -10.00, *p* < 0.001). No participant had HL classified as ‘inadequate’ after the nursing intervention. Verbal teach back method was the most frequent strategy used by nurses, and the use of a computer image was the most frequent visual aid.

**Conclusions:**

The use of a standardized HL intervention by nurses was shown to have a positive effect on patient general HL scores in specialty consultations in Spain. These results not only suggest that the use of a HL checklist can be an effective HL tool, but also reinforce the potential of nurses to make a positive impact on both individual and population health. Overall, these findings provide data that can be used by health systems, hospitals management, and nurse education programs to adopt strategies to improve patient HL and health outcomes, while potentially lowering costs and ineffective resource utilization related to inadequate HL.

**Supplementary Information:**

The online version contains supplementary material available at 10.1186/s12912-024-02447-1.

## Introduction

Health literacy (HL) is a multidimensional concept, addressing the resources and tools that people have to obtain, process, make judgements, manage, and act upon health information in ways that improve their health, wellbeing, and quality of life [1–3]. HL strategies are the actions that clinicians can use to ensure that patient health education is received and understood in meaningful, actionable, and empowering ways. The goal of HL teaching is that patients will be able to process, understand, make judgements about, ask informed questions, and act upon a variety of sources of health information received [1, 3].

Much has been written about the importance of health literacy (HL) among individuals and populations. Low HL has been linked to poor disease management, problems with medication adherence, and inconsistent use of preventative healthcare services, all leading to increased levels of morbidity and mortality [4–6]. Further, low HL imposes a burden on already struggling health systems due to patients’ increased use of emergency services, higher readmissions rates, and elevated costs related to health spending, disability payments, and loss of work and productivity [7–9].

In one retrospective study of 92,749 patients using the US Veteran’s Administration healthcare system over the course of three years, patients with inadequate or marginal HL cost the health system $143 million more than patients with adequate HL [10]. Another study examining the link between patients’ HL and their use of emergency services found that individuals with limited HL had significantly higher rates of emergency service use (OR: 1.57, 95% CI: 1.02–2.43) and were more likely to experience a potentially preventable hospital admission (OR: 1.65, 95% CI: 1.00-2.73) [11].

The scope of HL goes far beyond just teaching [12]. Clinicians should educate patients with the goal of equipping them to follow clinical instructions, adhere to prescribed treatments, and take actions to improve their health. There is agreement in health education literature that certain educational strategies are more effective than others in soliciting changes in patient behavior [13–15]. Some items cited as being more effective include the use of visual aids, inviting questions, demonstrations, summarizing and repeating instructions, teach back and show back techniques, the use of computers, and the use of multiple strategies instead of just one [16–20].

In particular, literature suggests that nurses play a pivotal role in the important quest of patient education and improved HL [21]. Nurses provide information about medication, how to care for lesions, and how to perform procedures such as giving oneself an injection of insulin [22]. They may also help patients understand how to obtain a doctor’s appointment, request a prescription refill, or know where to look for additional support such as social services. Nurses are often the communication interface between patients and doctors, and even act as mediators between patients and their family members or caregivers.

Researchers have approached the study of HL from various angles. Some researchers created and validated HL measurement instruments, and subsequently used them to measure HL in various countries and in distinct settings [1, 13, 23–30]. These efforts resulted in a body of information that has facilitated an important assessment of HL around the world, and especially in Europe. Another dimension of HL that has been examined is the effectiveness of different HL strategies, teaching techniques and methods [13, 18, 31–35]. Those studies are also of import, as they showed that certain HL interventions are more useful in changing patient behaviors and improving health outcomes [36].

While HL measurement tools have been created and used to calculate HL scores, and researchers have explored the effectiveness of specific educational strategies, few studies bring these two endeavors together. Further, we found scarce research that has done this before with a focus on the role of nurses. Therefore, the aim of this study was to examine the relationship between nurses’ use of a health literacy checklist and pre- and post-intervention HL scores, and the effects of frequency and types of HL strategies used.

## Methods

A quasi-experimental, non-randomized, pre-and-posttest method was used because it was a viable method to explore the effect of the nursing intervention on post-intervention HL scores. This method also enabled us to capture data about the frequency and types of HL strategies used by the nurses. The study ran for a period of 12 months, between April 2023–2024. It was approved on 16 March 2023 by the Department of Health, Ethical Research Committee of Aragón: PI23/083 following law 14/2007, 13 July for Biomedical Investigations and Applicable Ethics Principles. It was further approved on 23 February 2023 by the Aragones health services (salud). All participants were given a take home description of the study and signed consent forms.

### Selection of study sites

Top nursing management selected the four specialty consultations that would participate in the study. To select the sites, the number of patient visits per year to each type of consultation was examined. It was determined that there was a higher probability that patients would require three appointments within the 12-month study period at the diabetes, ostomy, cardiology, and digestive clinics. Therefore, these were the consultation types selected for the study.

### Selection and description of participants

The patient sample consisted of individuals who regularly attended specialty consultations in Zaragoza health sector III of the autonomous community of Aragon. Specialty consultations included: diabetes, ostomy, cardiology, and digestive. Inclusion criteria for the study were (1) age 18 or older, (2) willingness to complete the HLS_19_-Q12 survey two times (before and after HL interventions), and (3) the patient had the necessity to make three visits to the specialty consultation within the 12-month period of data collection. There were no other exclusions.

To obtain the sample group, the principal researcher followed a predefined weekly schedule of recruitment from April – November 2023. The principal researcher visited each specialty consult on predetermined days each week. The patients who had appointments on those days and satisfied selection criteria were invited to participate in the study. All 166 participants asked agreed to be in the study. After loss of patients due to missed appointments and invalid survey completion, before and after HL scores were calculated for 149 patients.

### Training of specialty clinic nurses

**Fig. 1:**
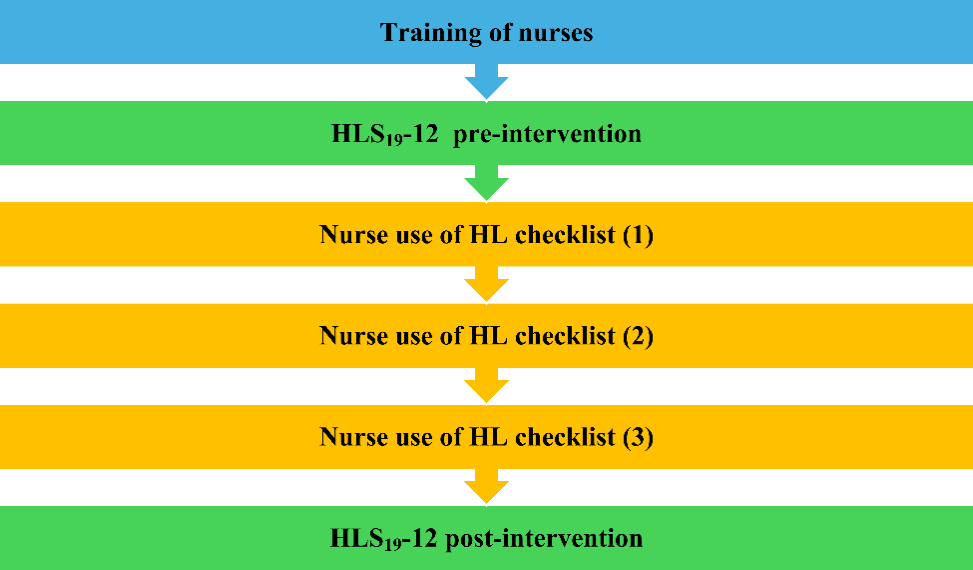
Process flow of the study design

A total of eight nurses participated in the study intervention. These nurses were chosen because they were the nurses already working in the chosen consultations. These nurses were considered to have advanced knowledge in their fields of practiced based on the fact that they had all passed exams for permanent positions, had more than 10 years of experience, and received specialized training for the medical condition they treat. In order to standardize the nursing intervention, the nurses participated in training. Each nurse agreed to attend the HL training course, consistently ask the questions on the HL checklist, use the strategies as instructed on thechecklist, and ensure that each patient enrolled in the study completed three visits to the consultation within a 12-month period (Fig. 1).

Nurse training was conducted by the principal researcher who was an advanced degree nurse. She also had vast professional experience as a patient and clinician educator, and was the project leader for an international healthcare quality standard that outlined requirements for HL [37]. Nurse training consisted of (1) an in person endorsement by the Director of Nursing highlighting the importance of the study for best clinical practice and patient outcomes, and an expression of her commitment and support of the study, (2) an overview of HL and why it is important, (3) a discussion of populations at risk for low HL levels, (4) a review of strategies clinicians could use to improve patients’ HL, (5) role play between nurses and pretend patients in hypothetical teaching situations, and (6) a description of the nurses’ roles and responsibilities in the study. Finally, nurses reviewed the content and wording of the HL checklist prior to its use. This was done to ensure that it was appropriate and understandable by both the nurses and the patient population they treated.

### Data collection and measurements

The health literacy survey named the HLS_19_-Q12 was used to assess general adult HL. The HLS_19_-Q12 had been validated internationally in 17 countries and in 17 languages using different types of data collection with acceptable psychometric properties and validity [30, 38]. It was further an attractive tool because it consists of only 12 questions, which minimizes the use of both patient and nurses’ time.

The HLS_19_-Q12 is a subjective, perception based instrument, that uses a four-point Likert scale to record patient’s perceptions concerning 12 health related items. Respondents were asked to rate the difficulty level of health related items by selecting “very difficult,” “difficult,” “easy,” “very easy,” or “I don’t know.” In accordance with Type P calculations, scores were calculated as the sum of the item’s numeric values scaled to a range from 0 to 100 [30]. If there were more than two invalid responses in a survey, that survey was disqualified from the study. Scores were then categorized based on the following scale: > 83.33 = Excellent, > 66.67 and ≤ 83.33 = Sufficient, > 50 and ≤ 66.67 = Problematic, and ≤ 50 = Inadequate [30].

The survey was completed during face to face consultations with patients. Reponses were captured on paper in one of the following ways: (1) by the patient alone, (2) an interview with the principal researcher, (3) patient with the help of a family member or caregiver, (4) patient with help of the principal researcher, or (5) by the specialty nurse. The HLS_19_-Q12 had been previously validated using the paper-assisted personal interview (PAPI) mode, so these were acceptable data collection methods [38]. The principal researcher was present for the completion of the surveys in case the patient had questions. All data was anonymized using a unique participant identifier.

The intervention in this study was the use of an HL checklist (Additional file 1). The checklist was created by the principal researcher based on three inputs. First, select items were chosen from the HLS_19_-Q12. Second, the teaching strategies included in the checklist were selected based on research suggesting that those were some of the most effective strategies. Lastly, studies suggest that the use of more than one teaching strategy is most effective. Therefore, the checklist also required this practice from the nurses [13, 18, 30, 39].

The checklist consisted of seven questions aimed at reinforcing the patients’ understanding of, and access to, health information. Three questions were open ended and allowed for continued dialogue between the patient and nurse if needed or desired. The checklist also required the HL strategy of summarizing or reinforcing the important points of that days’ appointment. The final section of the checklist required that the nurse use a minimum of two HL strategies listed in the checklist. For example, using the teach back or show back method along with the use of one or more visual aids. The checklist length and questions were chosen with the goal that its use would not require more than 10 min. This is key, given that both clinicians and patients are often short on time during consultations.

The nurses used the checklist with participants during all three of their appointments. The first use was after the initial HLS_19_-Q12 was completed by the patient. The second time was during the patient’s next regularly scheduled appointment. The final time was during the patient’s third visit. The patient also completed the post-intervention HSL_19_-Q12 at the end of the third appointment.

### Data analysis

IBM SPSS 27 was used to analyze survey data. To examine the difference between before and after HL scores, paired sample t-test and Wilcoxon tests were performed. Cohen’s d was used to determine effect size, with a d ≥ 8 considered to be a large effect. Frequency analysis was utilized to assess how often the nurses used each HL technique on the checklist. Pearson correlation determined which specific strategies as well as number of strategies had a statistically significant association with health literacy scores (HL scores) post-intervention. This study assumed a significance level of 0.05.

The Shapiro-Wilk test was used to test for normality before using paired sample t-test. Results were significant, so the null hypothesis of normal distribution was rejected. An outlier identified via boxplot was removed from the dataset, then the data assumed normal distribution.

## Results

### Patient heath literary scores pre- and post-nursing intervention

Paired sample t-test assessed the difference between HL scores pre- and post-intervention. The paired sample correlation test showed that the mean difference between the two scores was − 9.94, with a standard deviation of 11.50. The significance level indicated a statistically significant effect of the intervention on HL score (t = -10.00, *p* < 0.001). Cohen’s d was negative and 0.86, which indicated a large effect size, and that the mean HL score of the pre-intervention group was lower than the mean for the post-intervention group.

The Wilcoxon test was used to confirm the effect of the intervention. The mean score in HLS_19__Q12_group_1 was lower (2.39) than the mean score in HLS_19__Q12_group_2 (2.96). The z-statistic was − 7.12, and the asymptotic significance was < 0.001, showing a statistically significant difference in the scores of both groups. Based on mean ranks, it was proven that overall sample HL scores post-intervention were greater than pre-intervention.

The same HL score component was treated as a continuous variable for better understanding of the intervention’s outcome on patients’ HL scores. The results of the continuous variable (HLS_19__Q12 score) showed that pre-intervention, the mean HL score was 66.17 (SD:13.62), whereas post-intervention, the mean HL score improved to 75.89 (SD: 9.95).

**Table 1:** Statistical results for general HL scores pre- and post-nursing intervention

		Mean	Median	Std. Deviation	Std. Error Mean						
HLS_19_-Q12 score pre-intervention		66.17	66.67	13.62	1.09		**95% Confidence Interval of the Difference**			**Significance**	
HLS_19_-Q12 post-intervention		75.89	75.76	9.95	0.83		**Lower**	**Upper**	***t***	**One-Sided p**	**Two-Sided p**
Paired Differences pre-post intervention		-9.94		11.50	0.99		-11.90	-7.97	-10.00	*P* < 0.001	*P* < 0.001
					**Stder** ^**a**^	**Point Estimate**					
Paired Samples Effect Sizes				**Cohen’s** ***d***	11.50	-0.86	-1.06	-0.66			
HLS_19_-Q12 score pre- and post-intervention				**Hedges’** ***g***	11.57	-0.85	-1.05	-0.66			
	HLS_19_-Q12 score pre-and post-intervention Wilcoxon Signed Ranks Test									**Z**	**Asymp. Sig. (2-tailed)**
										-7.12^b^	*P* < 0.001

a. Standardizer. The denominator used in estimating the effect sizes. Cohen’s d uses the sample standard deviation of the mean difference. Hedges’ correction uses the sample standard deviation of the mean difference, plus a correction factor

b. Based on negative ranks

Most of the respondents in group 1 (pre-intervention) had HL that was ‘problematic’ equaling 48.7% (73 patients) of the valid sample, followed by 32.7% (49 patients) who had ‘sufficient’ HL. After HL interventions, most patients had HL that was ‘sufficient’ equaling 55% of the valid sample (82 patients) and 20.1% (30 patients) were ‘excellent’. In post-intervention group 2, only 24.8% (37 patients) scored ‘problematic,’ showing marked improvement. No participants had HL that was ‘inadequate’ after the nursing intervention (Table 2).

**Table 2:** Health literacy pre-and post-nursing health literacy intervention

Pre-intervention					
**Classification**		**N**	**Percent**	**Valid Percent**	**Cumulative Percent**
	Inadequate	16	9.6	10.7	10.7
	Problematic	73	44.0	48.7	59.3
	Sufficient	49	29.5	32.7	92.0
	Excellent	12	7.2	8.0	100.0
	Total	150	90.4	100.0	
Missing	System	16	9.6		
Total		166	100.0		
**Post-intervention**					
**Classification**		**N**	**Percent**	**Valid Percent**	**Cumulative Percent**
	Problematic	37	22.3	24.8	24.8
	Sufficient	82	49.4	55.0	79.9
	Excellent	30	18.1	20.1	100.0
	Total	149	89.8	100.0	
Missing	System	17	10.2		
Total		166	100.0		

**Fig. 2:**
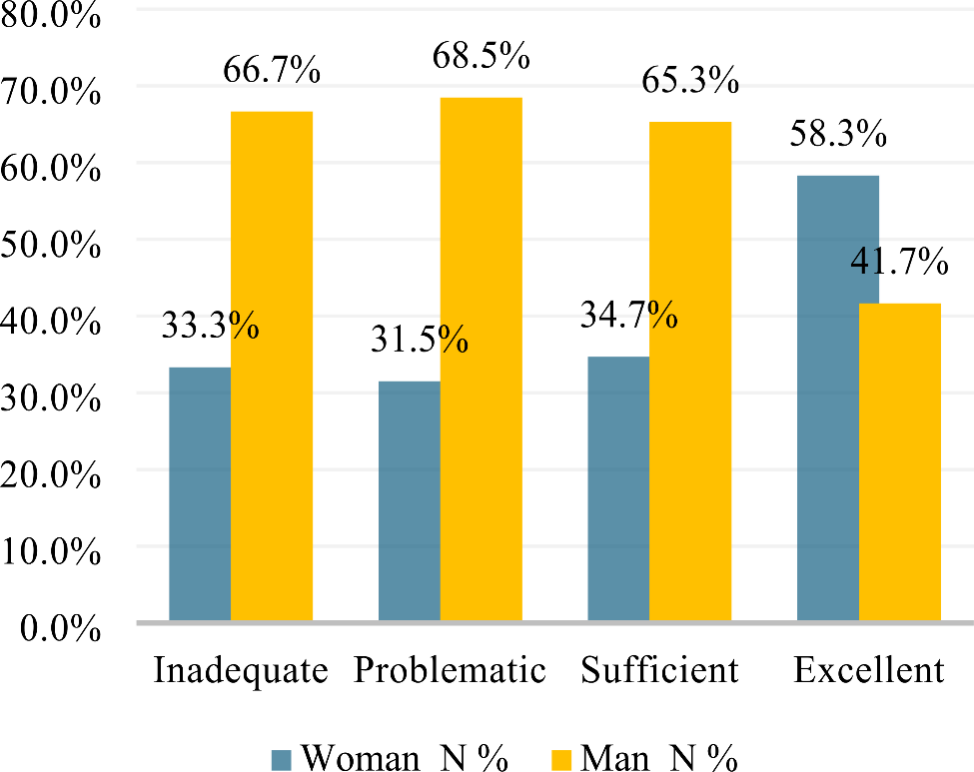
Percentage of participants by sex with inadequate, problematic, sufficient or excellent HL pre-intervention (cisgender)

**Fig. 3:**
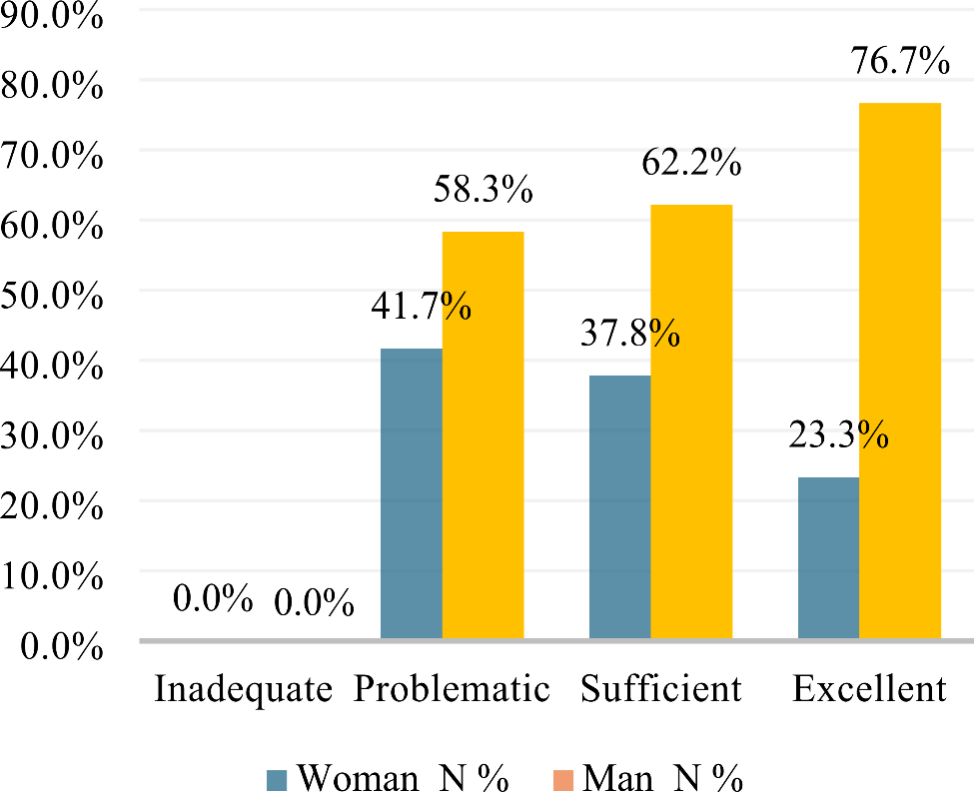
Percentage of participants by sex with inadequate, problematic, sufficient or excellent HL post-intervention (cisgender)

HL scores post-intervention were also analyzed by sex. All respondents identified as cisgender. For the pre-intervention HL survey, a greater percentage of men had ‘inadequate’ or ‘problematic’ HL than women. Women showed a greater percentage of ‘excellent’ HL than the lesser categories of ‘sufficient,’ ‘problematic,’ or ‘inadequate’. Post-intervention, the percentage of men whose HL was categorized as ‘excellent’ had increased from 41.7 to 76.7% (Figs. 2 and 3). Descriptive statistics showed that the mean HL score improved post-intervention for both women (68.07 to 75.45) and men (65.81 to 76.25).

### Frequency of health literacy strategies used by specialty practice nurses

Frequency analysis quantified the nurses’ use of specific strategies (tools, or techniques) chosen from the HL checklist. Verbal teach back method was the most frequent technique used over the course of the three appointments, selected by nurses 79% of the time. In regard to the section of the HL checklist that required the nurse to select a visual aid, use of a computer image was the most popular choice at 46%, followed by a physical model or device 31% of the time. The least frequently used verbal/action technique was the show back method (17%), and the least often selected visual aid was a poster (0.6%) (Table 3).

**Table 3:** Frequency of health literacy strategy used

	Appointment 1		Appointment 2		Appointment 3		Overall	
	Count	Column N %	Count	Column N %	Count	Column N %	Count	Column N %
**Verbal/Action**								
Teach back what you have learned (Verbal)	133	80.6%	126	76.4%	132	80.0%	391	79.0%
Show back what you have learned (Action)	37	22.4%	27	16.4%	20	12.1%	84	17.0%
None	19	11.5%	8	4.8%	4	2.4%	31	6.3%
**Visual Aids**								
Educational Brochure	38	23.0%	14	8.5%	10	6.1%	62	12.5%
Poster	0	0.0%	1	0.6%	2	1.2%	3	0.6%
Notes from the visit	10	6.1%	6	3.6%	11	6.7%	27	5.5%
Computer image	71	43.0%	81	49.1%	76	46.1%	228	46.1%
Picture on phone or app	2	1.2%	2	1.2%	7	4.2%	11	2.2%
Physical model or device	51	30.9%	55	33.3%	47	28.5%	153	30.9%
None	40	24.2%	18	10.9%	20	12.1%	78	15.8%

The study further examined the association between the total number of techniques used in all three appointments with the same patient and that patient’s HL score after the intervention. There was no statistically significant correlation between total number of techniques used from the checklist and HL score post-intervention.

### Relationship between the type of health literacy intervention used and patient health literacy score

Pearson correlation was used to determine whether any of the HL strategies used in the appointments had a statistically significant association with HL score post-intervention. The ‘show back what you have learned (action)’ technique had a statistically significant negative relationship with HL scores after intervention, *r* = -0.140, *p* < 0.1 (correlation is marginally significant). Moreover, the use of physical model or device visual aid also had a statistically significant negative link with HL scores post-intervention, *r* = -0.153, *p* < 0.1. There were no positive statistically significant associations revealed (Table 4).

**Table 4:** Correlations between type of health literacy strategy and post-intervention HL scores

		HLS_19__Q12p_score post-intervention
HLS_19__Q12p_score	Pearson Correlation	1
	Sig. (2-tailed)	
	N	148
Verbal teach back	Pearson Correlation	0.128
	Sig. (2-tailed)	0.120
	N	148
Action show back what you learned	Pearson Correlation	-0.140
	Sig. (2-tailed)	0.090
	N	148
Neither teach back nor show back	Pearson Correlation	-0.017
	Sig. (2-tailed)	0.840
	N	148
Educational brochure	Pearson Correlation	0.063
	Sig. (2-tailed)	0.449
	N	148
Poster	Pearson Correlation	-0.079
	Sig. (2-tailed)	0.342
	N	148
Notes from visit	Pearson Correlation	0.021
	Sig. (2-tailed)	0.804
	N	148
Computer image	Pearson Correlation	0.074
	Sig. (2-tailed)	0.371
	N	148
Picture on phone or app	Pearson Correlation	-0.011
	Sig. (2-tailed)	0.895
	N	148
Physical model or device	Pearson Correlation	-0.153
	Sig. (2-tailed)	0.063
	N	148
Did not use any visual aids	Pearson Correlation	0.026
	Sig. (2-tailed)	0.752
	N	148

**Correlation is significant at the 0.01 level (2-tailed)

*Correlation is significant at the 0.05 level (2-tailed)

## Discussion

This research examined the relationship between nurses’ use of a HL checklist and patient pre-and post-HL scores in specialty consultations in Spain. It further studied the effects of frequency and types of strategies used. Our data showed a statistically positive relationship between nurses’ use of the checklist and patient HL scores. In the present study the mean HL score was 66 before HL intervention, and post-intervention it increased to 76. In one of the first large scale studies of HL across 17 European countries using the HLS_19_-Q12, the mean HL score was 65 [30]. After the use of the checklist in this study, the mean HL score was notably higher than that of the larger European population previously studied.

Research suggests that individuals with moderate to high levels of HL are more likely to utilize preventive care, adhere to medical advice from doctors and nurses, take medications as prescribed, and effectively navigate the often complex healthcare system using available health information [6]. One promising outcome of this study is the likelihood that the participants who did experience an increase in HL will be able to benefit from these same positive health behaviors.

It is interesting to note that post-intervention, men had a larger increase in mean HL score than women. This could be influenced by the fact that women have historically been assigned the role of caregiver, and therefore they accumulate more knowledge around healthcare and health information over the course of their lives [40, 41]. In as such, their scores were higher than the men before the intervention, which left less room for improvement post-intervention.

In relation to types of HL strategies used, 79% of the time nurses chose the “teach back” technique. This technique has been proven to be an effective tool for increasing patient learning and clarifying misunderstandings [19, 42]. While statistics did not show a significant relationship between this technique and HL scores in this study, the combination of this technique with other items on the checklist could be the source of improved HL. In regard to visual aid,  “use of a computer image” was the tool most frequently used by the nurses in this study. Friedman et al. (2010) found that computers can be an effective health teaching strategy [18].

There were no statistically significant positive relationships found between the number or type of HL techniques used on HL scores. Nonetheless, the overall patient scores did improve significantly post-intervention. This suggests that the first part of the checklist, which contained the seven questions and one summarizing technique that allowed for open ended discussion and deeper dialogue may be key to engaging and educating patients. It also implies that the quality of the interaction is more important than the mere quantity of strategies used with the patient. Another possible explanation is that the HL checklist required the use of multiple strategies. This supports research reporting that the use of multiple strategies produces better learning and behavior outcomes [13, 16, 18].

Contact with nurses are some of the few relationships of trust that patients have during which they obtain and process heath information [43]. Hence, nurses can play a pivotal role in improving patient HL [44]. Our findings support previous work showing that nurses contribute significantly to support and advance patient HL [22]. A study in Spain found that patients receiving care from advanced practice nurses had higher satisfaction scores, felt they received more time and dedication in consultation, and received more information [45]. Likewise, our results show the value of nurses with specialized disease knowledge in increasing patient HL. In particular, the use of specialized and experienced nurses in this study emphasizes the potential benefits of promoting and formalizing the field of advanced practice nursing in Spain.

### Strengths and limitations

This study was novel in various ways. First, it looked at before and after intervention HL scores of a patient population. This is in contrast to many studies that set a baseline by measuring HL scores of a sample population, but do not perform interventions and reevaluate HL scores afterwards [27, 46]. The investigation was further important in that it not only used a new HL checklist, but sought to illuminate which HL techniques worked best within a patient population. The results of this study can be used to inform future exploration regarding which HL strategies are most effective in increasing HL.

Nurses use various standard assessment tools such as the Glascow Coma Scale, the Braden Scale, Morse Fall Scale, and the GAD-7 [47–49]. Our findings open a discussion concerning the need for a short, standardized tool for HL that clinicians can easily use with patients. The Agency for Healthcare Research and Quality’s Health Literacy Universal Precautions Toolkit is one such option [50]. However, the checklist in this research is shorter and specifically aimed at meeting the HL needs of the patient at the exact moment of patient, caregiver, or family member contact.

While this study used the checklist in specialty consultations, none of the questions were specific to any of the specialty consultations. Rather, the questions were general and could be used in all types of healthcare settings. Further, the list of HL strategy options on the checklist were varied and applicable in many settings, only depending on what resources are available to nurses at the time of interaction with the patient (e.g., computers, brochures, physical models, posters). Therefore, the creation of a rapid and feasible HL checklist based on the results from this study could be a viable tool for nurses to use in their daily interactions with patients and caregivers in a variety of care delivery settings.

Various study limitations should be considered when interpreting our findings. The HL checklist created for this project had not been validated. Therefore, future research involving validation of the checklist could strengthen these findings, while also providing an additional validated tool for use by researchers and clinicians. Further, there was no control group of patients who did not receive the nursing intervention. This makes it difficult to prove causality and lessens the certainty that the improvement in HL scores was not due to external factors that could have influenced the results. Another limitation was the inability to control for differences among the nurses and their teaching styles. Some nurses could have been more effective in using the checklist than others, and perhaps only their patients showed improvements in HL scores. Finally, the study took place in specialty consultations, and the results may not be generalizable to all care settings.

### Future research

While this study explored which HL interventions were statistically significant in improving HL scores in specialty consultations, further explorations should consider investigating teaching techniques that are most effective in various care settings [15]. For example, some techniques might work better in specialty consultations where there is more frequent follow up with the same patients, whereas others might be more impactful during the less-often general practitioner visit, or in a rapid setting like the delivery of emergency services.

According to the self-reported sociodemographic data, the profile of this sample population was significantly homogenous. In 2023 the OECD reported unprecedented amounts of new permanent immigrants and asylum seekers worldwide [51]. As countries around the globe are facing continued migration, health systems will need to provide care to increasingly diverse populations. In future studies, it would be valuable to explore whether certain HL strategies work better with patients from specific cultures and linguistic backgrounds. Similarly, nurses come from a diversity of backgrounds and ethnicities. These factors could influence the types of strategies used, and some nurses might not feel as comfortable using certain strategies based on cultural norms and power differentials. Therefore, this would be a valuable extension of our work.

## Conclusion

This study aimed to take HL research a step further, providing data that can be used by nursing education programs, as well as hospitals and health systems as they seek to create processes and protocols that increase service user (patients, families, and caregiver) HL. The results suggest that nurses’ use of a standardized checklist aimed at improving patient HL was effective. While some strategies on the checklist were shown to be more impactful than others, a mix of strategies appears to work the best. Nurses’ use of engaging questions, and summarizing what was discussed or learned during the appointment may be the techniques that most positively influenced HL scores in this patient population.

## Electronic supplementary material

Below is the link to the electronic supplementary material.

Supplementary Material 1

## Data Availability

Data is available upon request to the primary author.
