# Supplementary material for: The effect of nurse health literacy interventions on patient health literacy scores in specialty consultations: a quasi-experimental study
Source: BMC Nurs. 2024 Oct 25;23:786. doi: 10.1186/s12912-024-02447-1 (PMC11520141; doi:10.1186/s12912-024-02447-1)
Supplement: Supplementary file 1 — Supplementary Material 1 [file 12912_2024_2447_MOESM1_ESM.docx]

____________

**Health Literacy Checklist for Participating Patients**

**Consultation: Diabetes, Cardiology, Digestive, Ostomy (Circle one)**

Nurse: Patient Identification Number: Date:

| **Questions to ask at each visit** | **Asked?**  **Y or N** | **Nurse's Initials** | **Comments (if needed or desired)** |
| --- | --- | --- | --- |
| What are the questions you have about your health situation or medication? |  |  |  |
| What kind of additional information do you think might help you manage your situation? |  |  |  |
| **If there is a caregiver at the time of the consultation,** ask them (where appropriate) if they have questions or need more information about handling the situation. (Put NA if no caregiver is present) |  |  |  |
| Have you understood all the information from the doctor (if there has also been a consultation) and from me today? |  |  |  |
| Do you know what to do or where to go in case of an emergency? |  |  |  |
| How is your mood or emotions and how could I help you or recommend resources? |  |  |  |
| Do you use media as a source of health information, and if so, do you have any questions about what you've heard or read? |  |  |  |
| Summarize the most important points of that day's visit. Examples:  • Today we talked about . . .  • Now you know how to. . .  • Remember . . . |  |  |  |
| Do you understand all the follow-up instructions and details for your next appointment? **Make sure the patient has 2 more appointments scheduled** |  |  |  |
| **Actions to be taken on each visit** | **Completed**  **Yor N** | **Nurse's Initials** | **List the methods used and the visual aid used in the boxes below** |
| Choose at least one on each visit:   1. Teach back (Verbal) 2. Show back what you have learned (Action) |  |  |  |
| Use one or more visual aids   1. Educational Brochure 2. Poster 3. Notes from the visit 4. Computer Image 5. Picture on phone or app 6. Physical model or device |  |  |  |

**Document any notes or comments that may be helpful in improving patient care in the office (or in the SALUD health system in general):**
